# Biocomposites from Rice Straw Nanofibers: Morphology, Thermal and Mechanical Properties

**DOI:** 10.3390/ma13092138

**Published:** 2020-05-05

**Authors:** José Carlos Alcántara, Israel González, M. Mercè Pareta, Fabiola Vilaseca

**Affiliations:** 1 Advanced Biomaterials and Nanotechnology, Department of Chemical Engineering, Polytechnic School, University of Girona, 17003 Girona, Spain; jalcantarac@unitru.edu.pe; 2 LEPAMAP Group, Department of Chemical Engineering, University of Girona, 17003 Girona, Spain; israel.gonzalez@udg.edu; 3 Department of Architecture and Construction, Polytechnic University of Girona, 17003 Girona, Spain; mm.pareta@udg.edu; 4 Department of Fiber and Polymer Technology, KTH Royal Institute of Technology, SE-10044 Stockholm, Sweden; 5 Department of Industrial and Materials Science, Engineering Materials, Chalmers University of Technology, SE-412 96 Gothenburg, Sweden

**Keywords:** rice nanofibers, biocomposites, casting, mechanical properties, thermal properties

## Abstract

Agricultural residues are major potential resources for biomass and for material production. In this work, rice straw residues were used to isolate cellulose nanofibers of different degree of oxidation. Firstly, bleached rice fibers were produced from the rice straw residues following chemical extraction and bleaching processes. Oxidation of rice fibers mediated by radical 2,2,6,6-tetramethylpiperidine 1-oxyl (TEMPO) at pH 10 was then applied to extract rice cellulose nanofibers, with diameters of 3–11 nm from morphological analysis. The strengthening capacity of rice nanofibers was tested by casting nanocomposite films with poly(vinyl alcohol) polymer. The same formulations with eucalyptus nanofibers were produced as comparison. Their thermal and mechanical performance was evaluated using thermogravimetry, differential scanning calorimetry, dynamic mechanical analysis and tensile testing. The glass transition of nanocomposites was shifted to higher temperatures with respect to the pure polymer by the addition of rice cellulose nanofibers. Rice nanofibers also acted as a nucleating agent for the polymer matrix. More flexible eucalyptus nanofibers did not show these two phenomena on the matrix. Instead, both types of nanofibers gave similar stiffening (as Young’s modulus) to the matrix reinforced up to 5 wt.%. The ultimate tensile strength of nanocomposite films revealed significant enhancing capacity for rice nanofibers, although this effect was somehow higher for eucalyptus nanofibers.

## 1. Introduction

Biomass has become a subject of increasing research and debate over recent times due to its potential for energy and material production [1]. In fact, the current environmental concern is pushing the international community towards policies that aim to displace fossil resources by biobased ones. In this context, agricultural industries are playing a role since they use to produce huge amounts of crop wastes annually. Disposal of these wastes in landfills causes serious problems related to environmental contamination and harmful effects to human and animal health. Depending on the land use and management options, agriculture can be a source or a sink for atmospheric CO_2_ [2]. In general, the progressive increase in atmospheric concentration of CO_2_ and other greenhouse gases due to agriculture or industrial practices has created a worldwide awareness in identifying new strategies to solve this global problem.

Crop residues are defined as the nonedible plant parts that are left in the field after harvesting. Global production of residues from the six main crops (barley, maize, rice, soybean, sugar cane and wheat) is estimated to be of around 3.7 Pg dry matter yr^−1^, and this value could increase by 1.3 Pg dry matter yr^−1^ considering the current progress towards highly intensive agriculture [1]. The use of crop residues as a raw material is recommended, especially considering their chemical composition of lignocellulosic fibers, with significant amounts of cellulose in some cases. The mean values of some agricultural residues’ chemical composition are presented in Table 1.

The lignocellulosic character of agricultural residues makes them suitable as source of cellulose fibers, which are the most abundant biopolymers on earth. The hierarchical structure of cellulose fibers is unique, with linear β-1,4-glucan chains forming microfibrils of 3–4 nm diameter (about 30–40 chains), that bond together in bundles resulting in microfibers that intermix with hemicelluloses at the secondary and primary layers of the plant cell wall, with lignin mainly at the primary layer and middle lamella [8]. The extraction of cellulose from nanofibers and its application in composite materials is gaining relevance thanks to their inherent properties such as high strength and stiffness, light weight, biodegradability and renewability of their resources.

Nanocomposites are materials made up of at least two different phases where one component has some dimension in the nano range, typically below 100 nm or even below 50 nm. Structurally, nanocomposites are materials that involve nanosized filler particles (dispersed phase), a matrix (dispersion phase), and an interfacial region. The main advantage of nanocomposites compared to conventional ones is their unique characteristics due to their large surface area to volume ratio [9]. Therefore, nanocomposite materials take advantage of the outstanding properties of the nanosized dispersed phase. The extraordinary mechanical, thermal, and structural properties of cellulose nanofibers give them huge potential to act as a reinforcing agent. Besides, when strong interfacial adhesion with the matrix is attained, proper stress transfer from the matrix to the reinforcing phase is expected. Due to the hydrophilic nature of cellulose polymer, the interfacial region is crucial to allow good dispersion and distribution of nanocelluloses into the polymer matrix, and to promote good adhesion of the two components.

Poly(vinyl alcohol) (PVA) is the largest synthetic water soluble polymer produced in the world [10,11]. It is prepared by the hydrolysis of polyvinyl acetate. The degree of solubility, and the biodegradability as well as other physical properties can be controlled by varying the molecular weight (Mw) and the degree of hydrolysis (saponification) of the original polymer [11]. PVA possesses noticeable features such as water solubility, ease-of-use, film-forming property, and biodegradability [12]. Thanks to all these characteristics, poly(vinyl alcohol) (PVA) has been widely used for more homogeneous distribution of components in the preparation of blends and composites with several natural, renewable polymers like chitosan, nanocellulose, starch, or lignocellulosic fillers [10,11,12,13,14,15,16,17].

In this work, cellulose nanofibers from rice straw residues are obtained and used for the production nanocomposite materials. The performance of rice nanofibers will be compared to other well-known cellulose nanofibers from common sources like wood. Rice straw was first submitted to a chemical bleaching process to obtain rice cellulose fibers, followed by an oxidation mediated by radical 2,2,6,6-Tetramethylpiperidine 1-oxyl (TEMPO) as pretreatment to further extract rice cellulose nanofibers. Nanocellulose fibers from rice straw are tested as reinforcing element of poly(vinyl alcohol) polymer matrix. The hydrophilic character of the matrix is chosen as model for a fully favorable nanofiber–matrix interface in order to analyze the potential of rice cellulose nanofibers. The nanocomposites were prepared by casting to ensure proper dispersion and distribution of the nanofibers into the polymer. The thermal and mechanical properties of the nanocomposites are analyzed and compared to other currently used cellulose nanofibers.

## 2. Materials and Methods

### 2.1. Materials

Rice straw from the *Oryza sativa* species and of the appellation “Arroz de Valencia”, was used as the raw material to extract cellulose nanofibers for the further preparation of composite materials. Rice straw was air-dried for over 24 h and then cut into lengths of about 3–5 cm. Characterization of the raw material was performed according to the Tappi test methods (Tappi, 2003–2004): hot water extractives (T 207 cm-99), benzene-ethanol extractives (Tappi 204 cm-97), α-cellulose (Tappi 203 om-93), and Klason lignin content (Tappi T222 om-98). The holocellulose was determined by treating the extracted rice straw with NaClO_2_ solution. For comparison, cellulose nanofibers from eucalyptus were also used as polymer reinforcement.

Poly(vinyl alcohol) (PVA) from Sigma Aldrich (Madrid, Spain) was used as the polymer matrix. PVA, supplied as white powder, had a molecular weight of around 70000 g/mol and was 87–90% hydrolyzed.

For solvent extraction, ethanol, benzene and toluene were used. Bleaching of rice straw was performed with sodium chlorite (NaClO_2_) and acetic acid (CH_3_COOH). Further oxidation reaction of rice straw fibers was carried out using the radical 2,2,6,6-Tetramethylpiperidine 1-oxyl (TEMPO), sodium bromide (NaBr), sodium hypochlorite solution (NaClO) 15% *w*/*v*, sodium hydroxide (NaOH) and hydrogen chloride (HCl). For the evaluation of carboxyl groups, methylene blue in powder was used with a buffer solution made from boric acid (H_3_BO_3_) and potassium chloride (KCl). Finally, for the viscosimetric analysis, cupriethylenediamine (C_2_H_6_CuN_2_) was used as cellulose solvent. All chemical reagents were supplied by Sigma Aldrich and used as received.

### 2.2. Preparation of Rice Fibers

Rice straw was chopped using knives, milled, and meshed with a 40-mesh (400 μm) screen. The retained matter was submitted to solvent extraction with ethanol/toluene (40/60) for 24 h to remove pectin, waxes, and fats. After drying at atmosphere conditions, rice straw fibers were bleached as follows: 50 g of rice fibers were immersed in a solution containing 5 g of sodium chlorite (NaClO_2_) and 5 g of acetic acid (CH_3_COOH) in 1 L of distillate water. Stirring was maintained for 4 h at 80 °C. Rice fibers were afterwards thoroughly rinsed with distillate water. A second bleaching step was performed under the same chemical conditions for 2 h at room temperature. After proper rinsing, the rice fibers were kept at 4 °C in the fridge. The mean fiber length and diameter of the obtained rice straw fibers were analyzed by means of a MorFi Compact fiber analyzer from TECHPAP (Grenoble, France). For this purpose, a dilute suspension of fibers (25 mg/L) was analyzed using an optics and flow cell measurement. Data of more than 3000 fibers were analyzed and the fiber length distribution, diameter distribution, as well as the mean values, were obtained.

### 2.3. Preparation of Rice Nanofibers

TEMPO-mediated oxidation at basic conditions was used as pretreatment for the extraction of cellulose nanofibers. Rice straw fibers (1 g) were suspended in water (100 mL) in the presence of TEMPO radical (0.016 g, 0.1 mmol) and sodium bromide (0.1 g, 1 mmol). Sodium hypochlorite solution (NaClO) of 15% w/v was added in the suspension and the pH was adjusted to 10 by adding hydrogen chloride 0.1 M. Magnetic stirring was applied to the suspension in order to assure good dispersion of all the substances. The oxidation reaction started when the desired amount of NaClO was added into the system. In this case, different oxidation degrees were tested by adding 3, 5, 8, or 12 mmol of NaClO per gram of cellulose fiber. The addition was dropwise at room temperature with constant stirring of about 500 rpm. The pH was maintained constant at 10 by continuous addition of sodium chloride. The reaction finished when the pH was constant [18,19].

After oxidation, the cellulose fibers were centrifuged at 10,000 rpm for 10 min, to remove all the chemical reagents. The supernatant was discarded and the solids were resuspended in distillate water and centrifuged again. The process was repeated five times. The final suspension was 1% by weight. This suspension was disintegrated using and Ultraturrax IKA T24 digital working at 20.000 rpm during 5 min. The final appearance was a transparent gel-like suspension that was kept at 4 °C in the fridge for further use. This rice cellulose nanofibers were named r-CNF.

The TEMPO-mediated oxidation of cellulose fibers from eucalyptus fibers was carried out at neutral pH conditions according to methodology reported elsewhere [20]. The eucalyptus (hardwood) cellulose nanofibers were named h-CNF.

### 2.4. Characterization of Rice Nanofibers

Attenuated total reflectance Fourier transform infrared (ATR-FTIR) spectroscopy was performed on a Mattson Satellite spectrometer (Mettler Toledo, L’Hospitalet de Llobregat, Spain) equipped with a MKII Golden Gate Reflection ATR System. Spectra of the different oxidized rice cellulose nanofibers were recorded by co-adding 64 scans at 4 cm^−1^ optical resolution within the range 600–4000 cm^−1^. The samples were cut in about 5 × 5 cm and were immersed for 5 s in an HCl 0.1 M solution with the aim of acidifying the carboxylate groups.

The carboxyl content of oxidized fibers was measured by UV-visible spectroscopy with the methylene blue method [21,22]. Here, a weighted oxidized cellulose sample (approx. 10–15 mg) was suspended in 25 mL of aqueous methylene-blue chloride solution (300 mg/L) and 25 mL of borate buffer of pH 8.5. The suspension is stirred for 1 h at 20 °C in a 100 mL Erlenmeyer flask. After this time, the suspension is centrifuged at 10,000 rpm for 20 min to isolate the fibers. The supernatant contains the nonadsorbed methylene blue that is determined photometrically, employing a calibration plot. After centrifuging, 1 mL of supernatant is introduced in a 10 mL volumetric flask together with 1 mL of acid chloride HCl 0.1 M. The total amount of free, i.e., nonadsorbed, methylene blue was calculated from experimental results (A) using the UV-VIS Spectrophotometer Shimadzu UV 160 (Thermo Fischer, Bilbao, Spain), working at 664 nm wave length. The final amount of carboxyl groups in mmol per gram of cellulose is given by the equation:

(1) $$COOH\;\left(mmol/g\right)=\frac{\left(7.5-MB_{na}\right)\cdot 0.00313}{w}$$

where MB_na_ is the amount of nonadsorbed methylene blue (mg) and w is the dry weight of the sample (g).

The water retention value (WRV) was measured by separating a determined volume of cellulose nanofibers gel into two equal portions, which were then centrifuged in a Sigma Laborzentrifugen model 6 K15 at 2400 rpm for 30 min to eliminate nonbonded water. In order to retain the NFC, a nitrocellulose membrane with a pore diameter of 0.65 μm was used at the bottom of the centrifuge bottles. Once centrifuged, only the NFC in contact with the membrane was removed, weighed, and then dried at 105 ± 2 °C for 24 h in containers of previously measured weight. This methodology is based on Tappi um 256. The average WRV value was then calculated according to the next equation:

(2) $$WRV\;\left(\%\right)=\frac{\left(W_{w}-W_{d}\right)}{W_{d}}\cdot 100$$

where W_w_ is the wet weight (g) and W_d_ the dry weight (g).

The degree of polymerization (DP) was determined from intrinsic viscosity measurements, according to UNE 57-039-92 (which agrees with ISO 5351-1:1981) using cupriethylenediamine as solvent. The correlation between the intrinsic viscosity [η] and the degree of polymerization (DP) was calculated from the next equation:

(3) $$\left[\eta \right]=K{}^{\prime }\cdot DP^{a}$$

with K’ = 0.42 and a = 1 for DP < 950, and K’ = 2.28 and a = 0.76 for DP > 950 [23,24].

Original fibers were observed by scanning electron microscopy. For this, the used microscope was ZEISS DSM 960 (Zeiss, Jena, Germany) operating at 25 kV. Specimens were previously coated by sputtering 10 Nm gold at the surface. On the other hand, cellulose nanofibers were observed using transmission electron microscopy (TEM) by means of a ZEISS EM 910 Transmission-Electron Microscope (Zeiss, Jena, Germany). Samples were prepared by diluting the gel suspensions 10 times in distilled water. Later, 8 μL of the diluted nanocellulose suspensions were deposited on the membrane grid and, after drying, it was dyed with a 1% solution of uranyl acetate for 3 min. The surplus was removed with absorbent paper for the observation.

### 2.5. Preparation of PVA-Rice Nanocomposites

**Figure 1:**
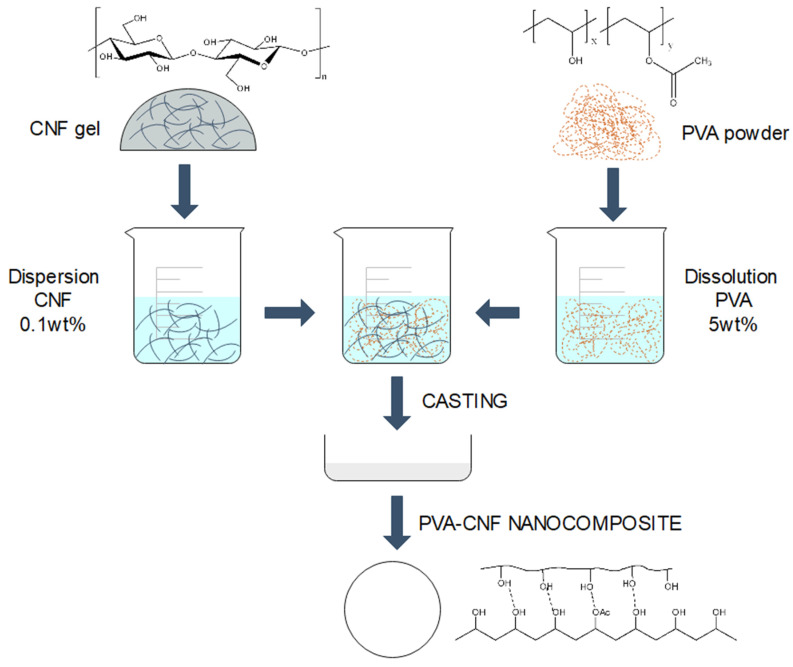
Illustrative scheme on the production mechanism of poly(vinyl alcohol)–cellulose nanofiber (PVA–CNF) nanocomposites.

For the preparation of nanocomposites, 12.5 g of poly(vinyl alcohol) (PVA) were dissolved in 250 mL of water at 10 °C in high magnetic stirring. The gel of cellulose nanofibers was diluted until 0.1 wt.%. Different amounts of cellulose nanofibers, from rice straw (r-CNF) or from eucalyptus (h-CNF), were mixed with the polymer solution in order to obtain nanocomposites comprising 0.5, 1, 2.5, and 5 wt.% of nanofiber reinforcement. Nanocomposite suspensions of 2 g dry weight were prepared. The PVA-nanocellulose suspensions were homogenized by stirring for 1 h, and cast in petri dishes afterwards. The suspensions were dried in an oven at 37 °C for four to five days. A transparent film was obtained from where the specimens were cut for material characterization. A scheme illustrating the production process of the PVA-CNF nanocomposites is presented in Figure 1.

### 2.6. Characterization of PVA-Rice Nanocomposites

Thermal characterization of the nanocomposites was conducted from differential scanning calorimetry (DSC) tests using Mettler Toledo DSC822 equipment (Mettler Toledo, L’Hospitalet de Llobregat, Spain). Around 7–9 mg of sample was placed in the 40 μL aluminum capsule. The temperature ramp was from 30 to 240 °C at a 10°/min heating rate under nitrogen flow of 40 μL/min. Values of glass-transition temperature (T_g_), melting temperature (T_m_), melting enthalpy (ΔH_m_), and degree of crystallinity (X_c_) were deduced from DSC analysis. Two heating/cooling procedures were applied.

Thermogravimetric analysis (TGA) was also performed using Mettler Toledo TGA851 equipment (Mettler Toledo, L’Hospitalet de Llobregat, Spain). For this, samples of around 10–15 mg were disposed in a 70 μL aluminum capsule. The assay was carried out from 30 to 650 °C at a 10°/min heating rate and under nitrogen flow of 40 μL/min. The degradation temperatures were obtained from the first derivative of the mass loss curve (DTG).

Prior to mechanical characterization, the specimens were conditioned in a Dycometal (Barcelona, Spain) climatic chamber at 23 °C and 50% relative humidity for 48 h before the tensile test and the dynamic mechanical analysis, according to ASTM D618 standard. Nanocomposite samples were mechanically tested in a universal testing machine Hounsfield model 42 (Tinius Olsen, Salfords, England) equipped with a 2.5 kN load cell. Sample dimensions were 5 mm wide and 45 mm long, and had a thickness of 0.2–0.3 mm. Preload was 0.1 N and cross-head speed was 5 mm/min. The results obtained were the average of at least five tested samples and data scattering within the range of 5–9%.

Dynamic mechanical analyses (DMA) of conditioned samples were performed using a Mettler Toledo DMA/SDTA861 instrument (Mettler Toledo, L’Hospitalet de Llobregat, Spain). The DMA measurements were done operating at tensile mode, at constant frequency of 1 Hz, amplitude of 20 μm and temperature range from −50 to 130 °C at a heating rate of 5°/min. The specimen dimensions were 5 mm width and 22.5 mm length, with thickness of 0.2–0.3 mm.

## 3. Results and Discussion

### 3.1. Rice Straw Fibers and Nanofibers

The valorization of agricultural wastes from rice crops is proposed in this study. In particular, we investigate the use of rice straw as potential raw material for cellulose nanofiber production. The chemical composition of the rice straw used in this study is shown in Table 2.

**Figure 2:**
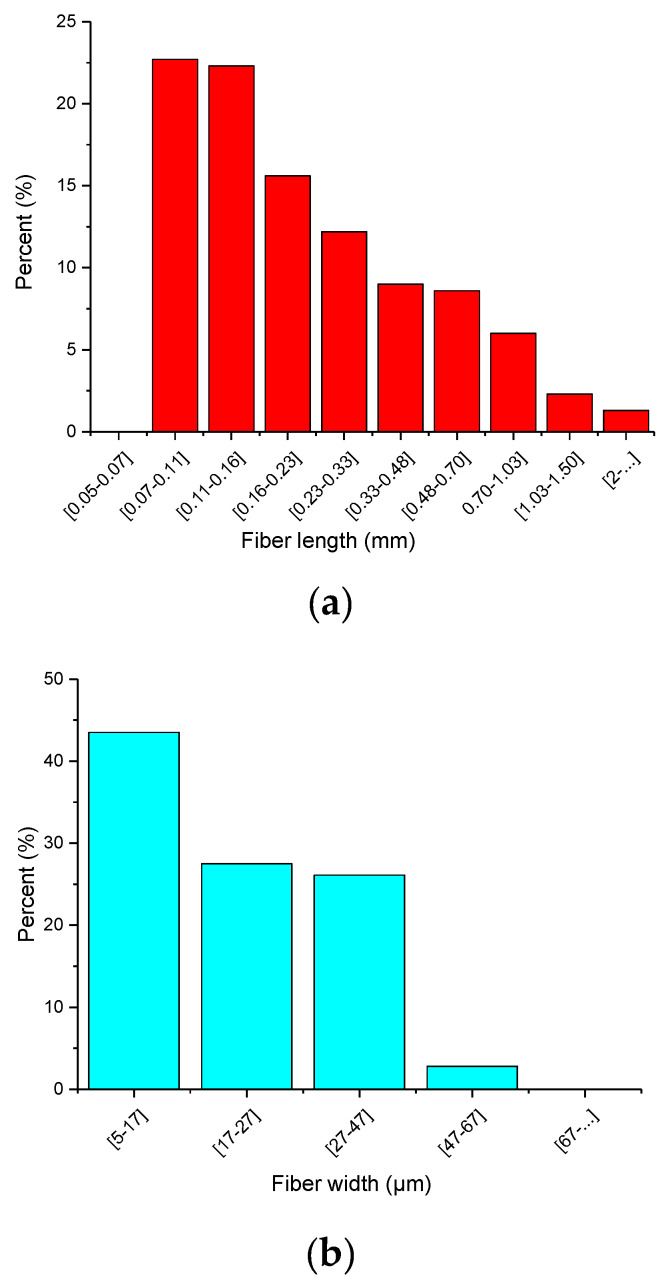
Arithmetic fiber length distribution (**a**) and fiber width distribution (**b**) of rice straw fibers.

These residues can be considered an interesting resource of cellulose fibers complementary to the other types of sources, mainly wood. The use of industrial residues or low-value byproducts as raw materials for the production of materials with high value, such as cellulose nanofibers, has been described in the literature by other authors [25,26,27]. It is worth noting that the holocellulose content (**α**-cellulose and pentosans) of rice straw residue is 60.7%. In general, the chemical composition is comparable to literature values, including other straw residues [4,5,27], with lignin values close to those for hardwood fibers [28]. Given the lower lignin content in the crop residues, along with their more porous structure compared to those of hardwoods and softwoods, milder pulping conditions with no sulfur processes can be applied for the lignin extraction [29]. In the current case, two bleaching processes with sodium chloride in acetic acid were employed to remove the lignin, once pectins and waxes were extracted. As a result, rice fibers were obtained with the length and diameter distributions shown in Figure 2. The mean weighted fiber length and fiber diameter were respectively 640 and 22,1 μm, with an aspect ratio of around 29. With this morphology, rice straw fibers can be considered as potential fibers for composite reinforcement.

**Figure 3:**
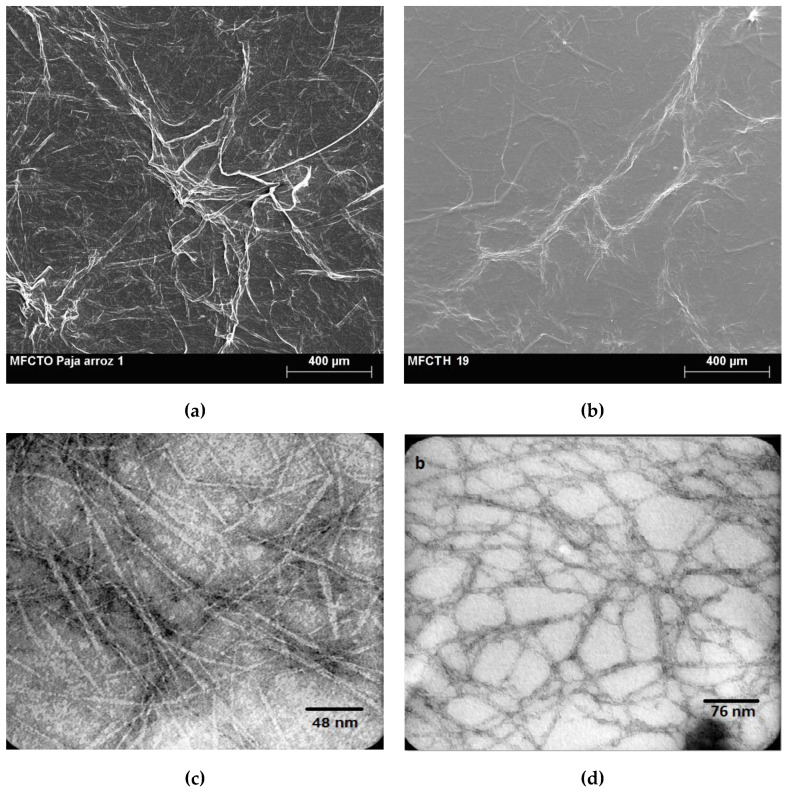
Scanning electron microphotographs of rice straw fibers (**a**) and of eucalyptus fibers (**b**). Transmission electron microscopy of rice straw nanofibers (**c**) and of eucalyptus nanofibers (**d**).

Cellulose fibers and nanofibers from wood are often used in comparison with the behavior of other natural fibers [30]. For the current study, micrographs from fibers and nanofibers from rice and eucalyptus are presented in Figure 3. An image of the obtained rice fibers observed by scanning electron microscopy is given in Figure 3a. As comparison, Figure 3b presents microphotography at the same amplification for bleached commercial eucalyptus pulp. Both the rice fibers and eucalyptus fibers were submitted to TEMPO-oxidation as pretreatment to isolate respective cellulose nanofibers, for which micrographs of transmission electron observation are presented in Figure 3c,d respectively. Mean nanofiber diameters were found to be 3–11 nm for rice nanofibers and around 4–6 nm for eucalyptus nanofibers, evidencing that both methodologies were suitable for extracting cellulose nanofibers. The fact that wood delivers somewhat thinner nanofibers is in agreement with results from other authors in the literature, also comparing wood and rice straw nanofibers [31].

Different oxidation degrees were used on the preparation of cellulose nanofibers from rice straw. Table 3 shows the main characteristics of TEMPO-oxidized rice nanofibers. The oxidation reaction was applied on four different extensions. Different amounts of sodium hypochlorite solution required different reaction times to complete the oxidation step. The resulting rice nanofibers showed different nanofiber lengths (polymerization degree) and different surface charges (carboxylic groups). In these terms, higher degrees of oxidation gave higher amounts of carboxylic groups, but lower cellulose chain lengths. This is expected considering the mechanism associated to TEMPO-oxidation reaction.

**Figure 4:**
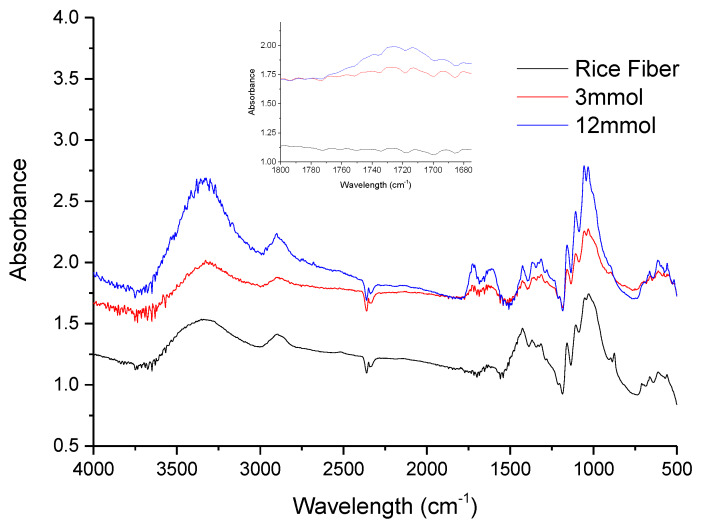
Attenuated total reflectance Fourier transform infrared spectroscopy (ATR-FTIR) spectra of rice fibers and for of nanofibers at two different degrees of oxidation.

FTIR is a rapid and nondestructive technique for the qualitative and quantitative determination of biomass components [32]. Moreover, FTIR with ATR (attenuated total reflectance) allows attenuation of the incident radiation and provides IR spectra without the water background absorbance. From ATR-FTIR analysis (Figure 4) it is confirmed the appearance of the absorption peak at 1720 cm^−1^ wavelength, associated to carbonyl groups. From the magnification of the ATR-FTIR spectra between 1680 and 1800 cm^−1^, rice fibers did not present any absorption band, while a peak appears after the oxidation reaction, more intense for higher extension reaction. In all the cases, the rest of the bands correspond to characteristic cellulose absorption peaks, as follows: –OH broad band between 3600 and 3200 cm^−1^ as well as the peaks at 1335 and 1205 cm^−1^; C–O–C bond at 1160 cm^−1^; CH_2_ stretching and bending vibrations at 2918, 3851, 1427 and 1315 cm^−1^; and finally bending vibration for CH at 1360 and 1280 cm^−1^.

**Figure 5:**
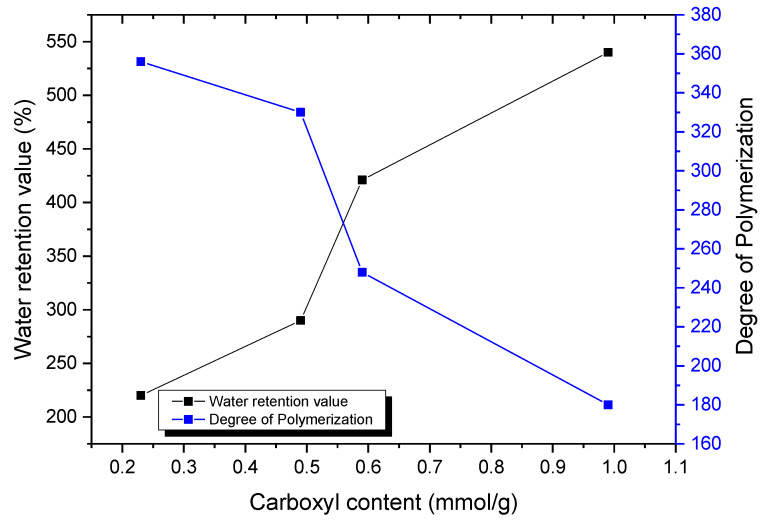
Water retention value and degree of polymerization with the carboxyl content.

The extent of the oxidation reaction impacted on the number of carboxylic groups on the cellulose surface (Table 3). In the current work, carboxylic groups were between 0,23 and 0,99 mmol per gram of rice cellulose fiber. These numbers agree with other values found in the literature for other cellulosic fibers, specifically for softwood [22]. It can be speculated that higher charge means more individualized nanofibers in suspension, although this has not been confirmed. It is clear though that the extent of the oxidation reaction, and so the number of carboxylic groups, influenced the water retention value of the resulting nanofibers. Water retention values relates to the amount of water adsorbed into the fibers. From the processing point of view, higher water retention values mean longer filtration times or larger casting times. Water retention values will combine with larger carboxylic content and also with larger fiber specific surface. Higher fiber individualization will favor the global specific surface and so the water retention values. The higher the oxidation extent, the lower the degree of polymerization was. The graphical relation between water retention values and polymerization degrees with the carboxylic content is illustrated in Figure 5. Then, fiber shortening was observed when the carboxylic content was higher. The polymerization degree was deduced from the viscous molecular weight analysis of cellulose fibers, using k’ = 0,42 and a = 1 as constant parameters for the current system [23,24]. There was a substantial decrease in the molecular weight of rice cellulose nanofibers with the extent of the oxidation reaction. As described previously, the viscous molecular weight was determined from dissolving cellulose nanofibers with cupriethylenediamine (CED). Some authors [18,33] have shown that the C6 aldehydes, formed as intermediate structures during the TEMPO-mediated oxidation, cause a remarkable depolymerization during dissolution in CED by β-elimination. The values found in our study agree with those found in the literature in similar experimental conditions [33].

### 3.2. PVA–Rice Nanocomposites

The combination of rice cellulose nanofibers with poly(vinyl alcohol) (PVA) allows PVA–rice nanocomposites to be obtained. Since both components are either water-soluble (polymer) or water-dispersible (rice nanofibers), casting in water was the chosen methodology to ensure proper distribution and dispersion of the reinforcing nanofibers into the polymer matrix and to avoid nanofiber aggregation. One important advantage of using PVA is the polar affinity of the polymer chains with cellulose fibers and nanofibers. It is therefore used as model system in order to analyze the reinforcing effect without side effects due to interface compatibility. Moreover, water-based procedures will avoid nanofiber aggregation during nanocomposite formation, which is one of the main issues in this field. In order to be able to compare the performance of rice nanofibers with others commonly used, PVA nanocomposites reinforced with cellulose nanofibers from eucalyptus (hardwood) were also analyzed.

The thermal characteristics as well as the crystallinity level of the polymer in nanocomposites are summarized in Table 4.

Glass transition temperature was determined from the second heating process applied to the samples. In the first heating process, the peak collided with the presence of water in the sample. Water integration gave 1.48% of water in the PVA sample, and between 0.31 and 0.35% of water in the biocomposites. Only the dry weight of the samples was used in further DSC calculations. In all cases, the addition of either nanofiber into the matrix moved the glass transition towards slightly higher temperature values. The same occurred with maximum melting temperature: biocomposites showed higher melting temperature than unreinforced matrix. This was more evident from the second heating step. In regard to the degree of crystallinity, the nanofibers did not act as nucleating agent, at least from the sample preparation (first heating, $\chi_{c}^{a}$). Instead, from the second heating ($\chi_{c}^{b}$), rice nanofibers did promote polymer crystallinity, although this was not observed for the eucalyptus nanofibers. The different nanofiber diameter and morphology of rice and eucalyptus nanofibers can explain this difference. Rice nanofibers were more rigid that eucalyptus nanofibers, better acting as nucleating agents.

**Figure 6:**
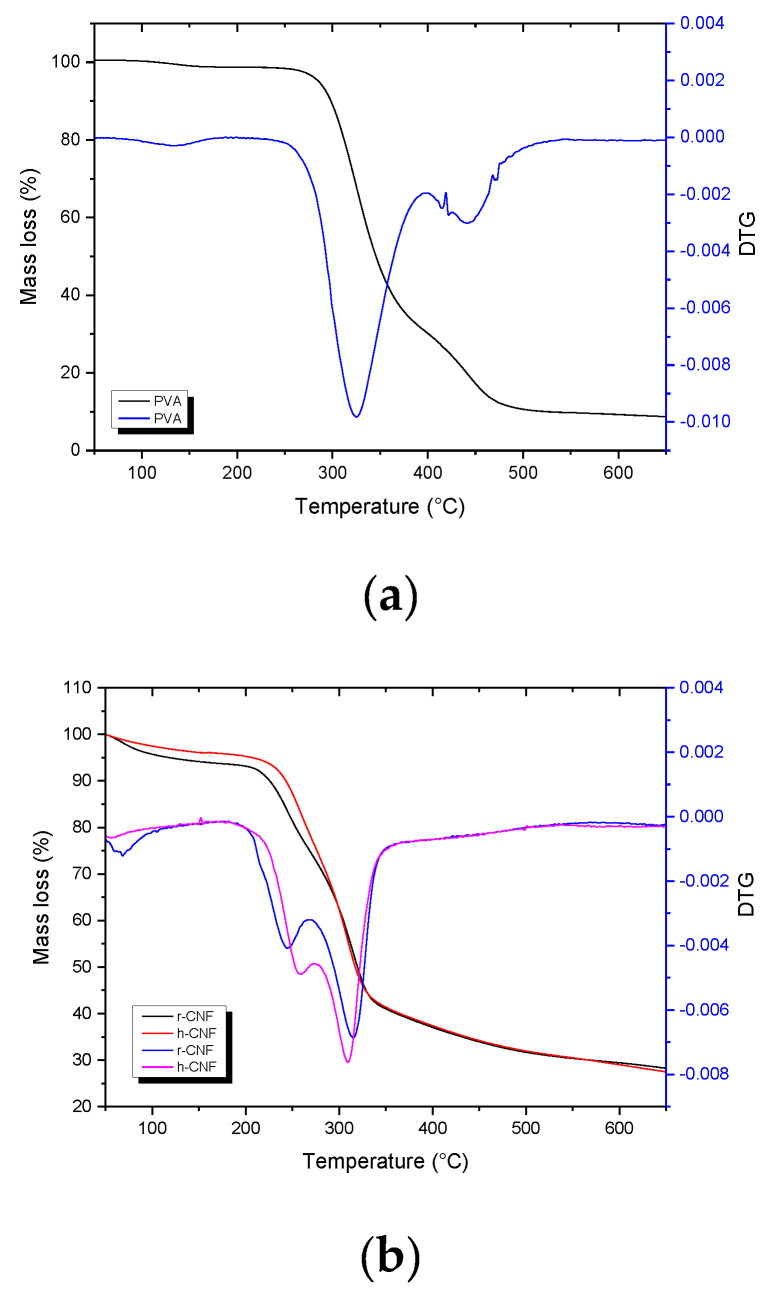

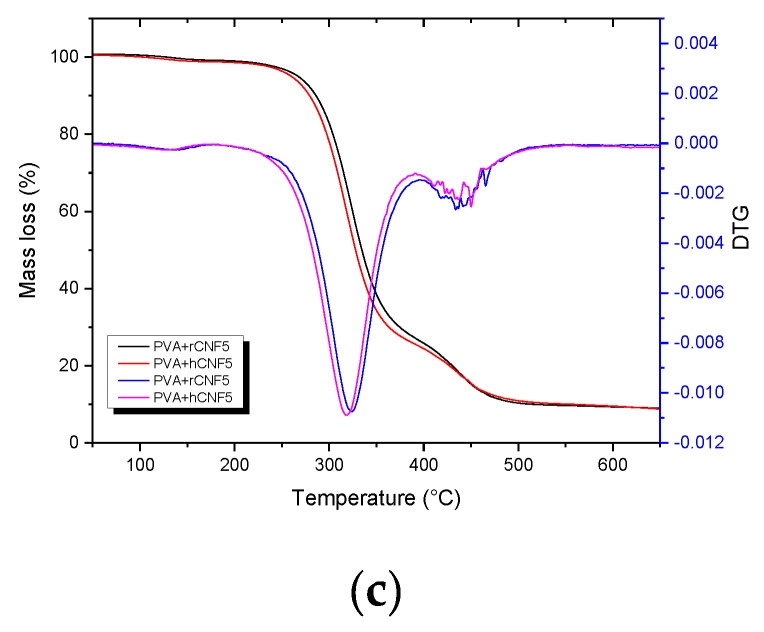
Mass loss (%) and first derivative (DTG) of polyvinyl alcohol (**a**), rice nanofibers (r-CNF) and hardwood nanofibers (h-CNF) (**b**), and of the respective nanocomposites containing 5 wt.% of nanofibers (**c**).

Thermal stability of biomaterials is important for their applicability in biocomposite fields. Figure 6 displays the TG and the differential thermogravimetric curves of the net polymer (Figure 6a), both nanofibers (Figure 6b), and of the respective PVA nanocomposite at 5 wt.% nanofiber content (Figure 6c). PVA polymer showed two main degradation peaks at 339 and 458 °C. Our polymer was 90% hydrolyzed. The literature shows that fully hydrolyzed PVA also shows two main degradation peaks (at 375 and 440 °C), corresponding to the chain-stripping produced by the removal of water molecules (dehydration of the PVA polymer) followed by chain scission and decomposition [34]. For partially acetylated PVA like in the current case, the first peak shifted towards lower temperatures.

The mass loss around 150 °C accounts for volatiles and additives present in the matrix. Two main degradation bands are also found in both types of nanofibers, one around 260–270 °C and another at 325–330 °C (Figure 6b). The first corresponds to hemicelluloses and the second to cellulose itself. The deviation from linear in the curves below 200 °C stand for the slow loss of water molecules kept inside the rice or eucalyptus nanofibers’ structure. Once inside the biocomposite, the hemicellulose band was not visible, and only the main peaks from cellulose and the polymer were present (Figure 6c).

**Figure 7:**
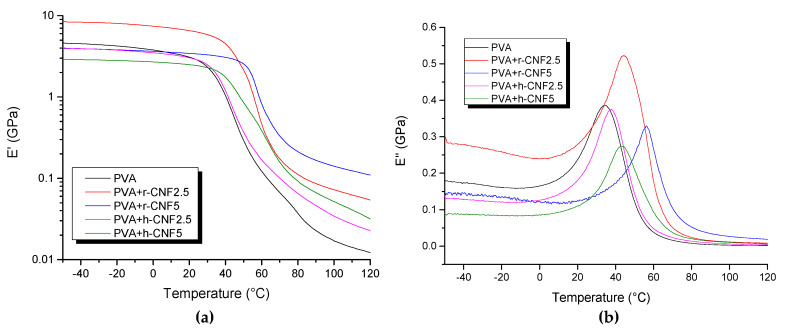
(**a**) Change in storage modulus (E’) and (**b**) loss modulus (E’’) as function of temperature for PVA and biocomposites at 2.5 and 5 wt.% content of rice nanofibers (r-CNF) or eucalyptus nanofibers (h-CNF).

Mechanical properties of biocomposites were investigated from DMA and tensile tests. The variation in storage modulus with the temperature of rice and eucalyptus bionanocomposites is presented in Figure 7. Reinforcing of PVA with nanofibers favored a higher storage modulus above room temperature and especially beyond glass transition (rubbery region). Reinforcing with these nanofibers is beneficial when working at higher temperatures. In the glassy region, only rice nanofibers produced some benefit compared to the polymer. Quan et al. also proved the increase in storage modulus for PVA–cellulose nanofiber composites above 30 °C as compared to the plain matrix [35]. It was reported that the difference between the elastic tensile modulus of cellulose nanofibers and that of the matrix is not high enough to benefit from a reinforcement effect in this temperature range. From our study, however, rice nanofibers provided a better reinforcement effect than eucalyptus nanofibers at both glassy and rubbery regions. We do not have a clear explanation for this, but it could be related to the different morphology of both types of nanofibers. Hence, while eucalyptus nanofibers are more flexible, rice nanofibers have a more rigid shape, as seen in the TEM images from Figure 3.

Other authors also found little influence on the storage modulus in eucalyptus CNF nanocomposites, although the methodology for composite preparation was different, and they claimed aggregation of their nanofibers [36]. Values of Tg deduced from the peak of the loss modulus (also in Figure 7) proved the different influence of both type of nanofibers. The dynamic loss modulus is often associated with internal friction and is sensitive to different kinds of molecular motions, relaxation processes, transitions, morphology, and other structural heterogeneities. The determination of the glass transition temperature (Tg), from the loss modulus, gave 35 °C for the net PVA, and 43 and 58 °C for rice nanocomposites at 2.5 and 5 wt.%. Instead, the same formulations with eucalyptus nanofibers gave tan δ of 40 and 42 °C, respectively, so higher than the matrix but below that for rice nanofibers.

**Figure 8:**
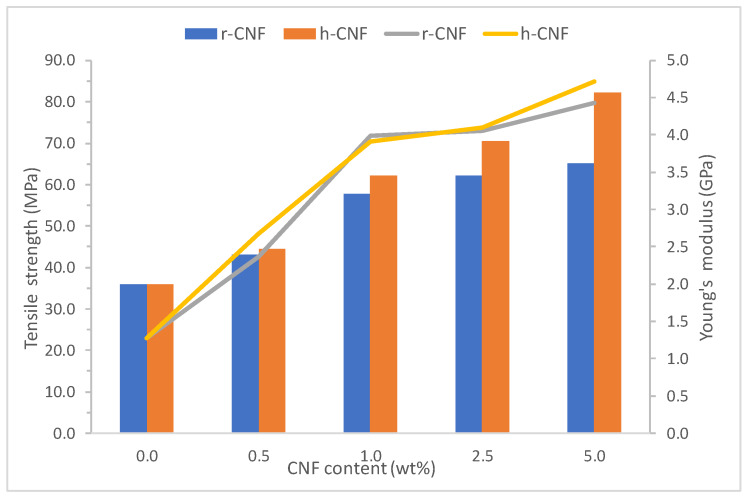
Graphical representation of tensile strength and Young’s modulus for all bionanocomposites.

The properties from tensile tests of PVA and all PVA biocomposites up to 5 wt.% of rice nanofibers or eucalyptus nanofibers are presented in Table 5. Graphical representation is in Figure 8. The same formulations of rice cellulose nanofibers (r-CNF) and eucalyptus nanofibers (h-CNF) were prepared for comparison. The net PVA matrix was more soft and weaker than the biocomposites, but with tensile strength and Young’s modulus comparable to high-density polyethylene or polypropylene. This is good since any improvement will make competitive materials, in terms of mechanical behavior. For biocomposites with rice CNF, mechanical properties, namely tensile strength and Young’s modulus, showed a linear increase up to 1 wt.% nanofiber content, and then the increments were less pronounced. Thus, incorporation of 1 wt.% r-CNF gave 1.6 times ultimate tensile strength, whereas incorporation of up to 5 wt.% r-CNF produced only 1.8 times greater ultimate tensile strength, both compared to the plain PVA matrix. Cellulose nanofibers from eucalyptus pulp always produced major improvements, especially for the tensile strength, which were 1.7 and 2.3 times higher for the same respective formulations. The higher increments on the tensile strength for the eucalyptus nanofibers can be explained due to the nanofiber morphology and their lower diameter compared to rice nanofibers. However, both types of nanofibers performed similarly for the Young’s modulus with similar increments. It was noticeable that there was a more than 3.5 times increase in rigidity with only 5 wt.% CNF. As expected, elongation at break decreased with the amount of CNF. However, deformations for biocomposites from eucalyptus nanofibers were larger compared to those from rice nanofibers. It seems, therefore, that the different morphology of eucalyptus nanofiber was beneficial for higher elongations at break.

## 4. Conclusions

In this work, cellulose nanofibers were extracted from rice straw, showing that high-added-value products can be obtained from agricultural residue. This residue contains more than 60% holocellulose, of which about 41% is α-cellulose. Rice fibers were produced following bleaching processes and the obtained fibers had an aspect ratio of 29. From the following procedure, rice nanofibers with diameters ranging between 3–11 nm were extracted. Rice nanofibers were used to reinforce poly(vinyl alcohol) matrix by casting. For comparison, eucalyptus nanofibers were also used to produce nanocomposites. The reinforcing capacity of rice nanofibers was proved, increasing by 3.5 times the Young’s modulus of the polymer. Similar stiffening was found for eucalyptus nanofibers. Instead, eucalyptus nanofibers were more favorable in strengthening, with ultimate tensile strengths 2.3 times higher than that from rice nanofibers, with ultimate strength 1.8 times that of the matrix, all values for 5 wt.% nanofiber content. The different morphology of both nanofibers behaved differently with polymer deformation or polymer crystallinity. Higher flexibility for eucalyptus nanofibers resulted in higher biocomposite deformation, but less ability to act as nucleating agent for crystal growing. Conversely, rice nanofibers acted distinctly as a nucleating agent for polymer crystal growing. Similarly, at DMA tests, rice nanofibers were more favorable to keep the storage modulus at a rubbery state, and clearly increased the Tg much more, as determined from the loss modulus peak.

All theses findings should encourage the use of agricultural residues as biomass for added-value materials and help in recommending better solutions for crop waste.

## Figures and Tables

**Table 1:** Chemical composition of agricultural residues.

Agro-Industrial Waste	Chemical Composition (% w/w)				Ref.
	Cellulose	Hemicellulose	Lignin	Ash	
Sugarcane bagasse	30.2	56.7	13.4	1.9	[3]
Rice straw	36.2	23.5	15.6	12.4	[4]
Corn stalks	61.2	19.3	6.9	10.8	[3]
Sawdust	45.1	28.1	24.2	1.2	[3,5]
Sugar beet waste	26.3	18.5	2.5	4.8	[3]
Barley straw	33.8	21.9	13.8	11	[6]
Cotton stalks	58.5	14.4	21.5	10	[6]
Oat straw	39.4	27.1	17.5	8	[5]
Soya stalks	34.5	24.8	19.8	10.4	[7]
Sunflower stalks	42.1	29.7	13.4	11.2	[7]
Wheat straw	32.9	24.0	8.9	6.7	[5,6]

**Table 2:** Composition of rice straw % (w/w dry weight).

α-Cellulose	Pentosan	Klason Lignin	Benzene-Ethanol Extractives	Hot-Water Extractives	Ashes
41.2	19.5	21.9	0.56	7.3	9.2

**Table 3:** Characteristics of TEMPO-oxidized rice nanofibers.

Amount of NaClO (mmol/g)	Oxidation Time (min)	Water Retention Value (%)	Carboxylic Groups (mmol/g)	Degree of Polymerization DP	Viscous Molecular Weight (g/mol)
3	110	220	0.23	356	57,600
5	140	290	0.49	330	48,600
8	190	421	0.59	248	40,300
12	220	540	0.99	180	29,200

**Table 4:** Thermal characteristics from DSC analysis. Glass transition temperature ($T_{g}$), Melting temperature ($T_{m}$), Melting enthalpy ($\Delta H_{m}$), and degree of crystallinity ($\chi_{c}$) of the polymer. Superscript **a** is for the first heating process, superscript **b** is for the second heating process. ($\chi_{c}\left(\%\right)$ = ($\Delta H_{m}^{}$)/(($\;\Delta H_{m}^{0})$ × ω))*100), with $\Delta H_{m}^{0}$ is the heat of fusion for the 100% crystalline polymer, which is estimated to be $\Delta H_{m}^{0}$ = 139 J/g for PVA-88 hydrolyzed; ω is the weight fraction of polymeric material in the respective composites).

Sample	$T_{g}^{b}$ (°C)	$T_{m}^{a}$ (°C)	$T_{m}^{b}$ (°C)	$\Delta H_{m}^{a}$ $\left(J\cdot g^{-1}\right)$	$\Delta H_{m}^{b}$ $\left(J\cdot g^{-1}\right)$	$\chi_{c}^{a}$ (%)	$\chi_{c}^{b}$ (%)
PVA	67.9	193.2	167.8	54.69	30.47	39.3	21.9
PVA/r-CNF2.5	69.0	194.8	176.9	27.73	30.84	19.9	22.2
PVA/r-CNF5	69.3	194.8	178.7	28.36	35.85	20.4	25.8
PVA/h-CNF2.5	69.7	194.9	171.1	27.22	25.66	19.6	18.5
PVA/h-CNF5	70.2	194.3	175.4	26.23	25.02	18.9	18.0

**Table 5:** Main tensile properties of PVA and bionanocomposites ($E_{Young}:\;$ Young’s modulus, σ:  ultimate tensile strength and $\varepsilon_{break}:\;$ elongation at break).

Sample	$E_{Young\;}\;(\mathrm{GPa})$	***σ***(MPa)	$\varepsilon_{break}(\%)$
PVA	1.27 ± 0.1	35.9 ± 1.5	136.5 ± 10.5
PVA/r-CNF0.5	2.37 ± 0.3	43,1 ± 2.0	105.1 ± 5.2
PVA/r-CNF1	3.99 ± 0.1	57.8 ± 1.3	2.54 ± 1.7
PVA/r-CNF2.5	4.05 ± 0.1	62.1 ± 2.0	1.90 ± 1.2
PVA/r-CNF5	4.43 ± 0.2	65.1 ± 1.1	1.76 ± 0.5
PVA/h-CNF0.5	2.67 ± 0.2	44.6 ± 2.9	128.8 ± 7.7
PVA/h-CNF1	3.91 ± 0.1	62.1 ± 2.0	2.99 ± 2.5
PVA/h-CNF2.5	4.10 ± 0.1	70.6 ± 2.1	3.42 ± 1.3
PVA/h-CNF5	4.72 ± 0.2	82.2 ± 1.4	3.66 ± 0.8
